# Restless Legs Syndrome and Poliomyelitis: New Evidences of an Old Observation?

**DOI:** 10.3389/fneur.2015.00023

**Published:** 2015-02-10

**Authors:** Andrea Romigi, Mariangela Pierantozzi, Francesca Izzi, Maria Teresa Desiato, Claudio Liguori, Angela Marchi, Nicola B. Mercuri, Fabio Placidi

**Affiliations:** ^1^Neurophysiopathology Unit, Department of Systems Medicine, Sleep and Epilepsy Medicine Centre, Tor Vergata University and Hospital, Rome, Italy; ^2^IRCCS Neuromed, Pozzilli, Italy; ^3^Neurology, Department of Systems Medicine, Tor Vergata University and Hospital, Rome, Italy; ^4^Clinica Neurologica Ospedale S. Eugenio, Rome, Italy; ^5^IRCCS Santa Lucia Foundation, Rome, Italy

**Keywords:** restless legs syndrome, poliomyelitis, fatigue, post polio syndrome, sleep

The discussion of this research paper may represent the starting point of the debate about relationship between restless legs syndrome (RLS) and poliomyelitis. The recent paper published by Kumru et al. (1) found a high frequency of RLS (40.4%) in consecutive unselected patients affected by sequelae of poliomyelitis in an uncontrolled setting. In addition, they showed responsiveness to dopaminergic treatment of RLS in these patients. Very recently, we published the first case–control survey investigating the real magnitude of RLS, fatigue, and somnolence in postpolio syndrome (PPS) and its impact on quality of life (2). Patients taking medications potentially interfering with RLS, with RLS familial history or affected by other medical conditions predisposing to RLS were excluded in order to avoid confounding factors. We evaluated 66 PPS patients and 80 healthy controls. We found a significant higher prevalence of RLS in PPS patients (63.6%) than in healthy controls (7.5%). Interestingly, fatigue severity (FSS score) was higher in PPS affected by RLS than those unaffected (*p* = 0.03) and statistically correlated with RLS severity. In addition, an overall significant decrease of quality of life (SF-36 scores), including Physical Function, Physical Role, and Bodily Pain domains, was also found in PPS patients with RLS when compared with those not reporting RLS symptoms. Most of SF-36 items (Physical Role, General Health, Vitality, Social Functioning, Role Emotional, and Mental Health) were negatively correlated with RLS severity. We did not find a relationship between RLS severity and muscle weakness severity in PPS. Our study is in line with the results obtained by Kumru et al. (1) confirming that RLS is frequent in patients with poliomyelitis sequelae. This link was firstly observed several years ago. In 1947, Luft and Muller (3) reported RLS in poliomyelitic patients, and they stated that “*symptoms were more prominent in the extremities that have been attacked by poliomyelitis*” hypothesizing a “*vasomotor disturbance*.” In 1950, Perdrup (4) described a single case of “*a patient with sequelae after poliomyelitis, attacks of restless legs accompanied by ulcer formation*.” This author clearly described that “*the pains are worst when she is warm in the bed, much less severe when she is up, and the air is cold*.” The intensity was so severe that “*she has asked to have the leg amputated*.” In the early 1940s, Sister Elizabeth Kenny, an Australian nurse, attempted to revolutionize the treatment of polio, introducing the concept of the need to exercise muscles affected by polio instead of immobilizing them reporting improvement of polio symptoms and fatigue especially for children who were restless (5). We may hypothesize that exercise also improved RLS symptoms in such patients with sequelae of poliomyelitis avoiding worsening during rest and inactivity.

After 60 years only isolated reports of RLS occurring in PPS were reported, although circadian fatigue, PLMS, and sleep complaints were frequently described (2, 6). Therefore, the findings from Kumru et al. (1) and our data (2) represent the first attempt to demonstrate a biological plausibility for a link between poliomyelitis sequelae and RLS. More difficult is to clarify the reason of this association. Interestingly, Kumru et al. (1) hypothesized that the reduced mobility of PPS patients may represent a clinical equivalent of the diagnostic “Suggested Immobilization Test” predisposing to RLS. On the other hand, the sequelae of polio, similarly to other spinal cord RLS-related diseases, may induce an anomalous sensorimotor integration at the spinal interneuronal level. This phenomenon may be mediated by dopaminergic deficit and/or to loss of supraspinal inhibitory influences (2). The diencephalon–spinal dopaminergic pathway, which projects its terminal innervations in the dorsal horn at all spinal levels, may represent a hypothetical common anatomical substrate involving RLS and PPS (2).

Another link may be represented by inflammatory mechanisms that involve more than 90% of neurological conditions associated with RLS and that have been supposed to play a role also in primary RLS (7). This hypothesis may explain the growing evidence that the fatigue affects several CNS regions and pro-inflammatory cytokines (i.e., IL-1, IL-6, and TNF-alpha) that are released during systemic inflammation similarly to RLS and PPS (8).

Fatigue is a key symptom of PPS, marked by a progressive course, a widespread distribution, and able to affect significantly quality of life mostly when associated with RLS (2). In addition, fatigue in PPS may be characterized by circadian changes, resembling the circadian pattern of RLS symptoms (6). In conclusion, we can consider PPS as a possible cause of a secondary form of RLS associated with both fatigue and quality of life. RLS affect fatigue and quality of life in PPS showing a significant correlation with RLS severity (2) and RLS symptoms were reported as more disturbing in the affected than in the less-affected or non-affected leg by 90% of PPS with RLS (1). The responsiveness of RLS in patients with poliomyelitis sequelae to dopaminergic treatment demonstrated by Kumru et al. (1) should induce to test the possible effect of RLS treatments on PPS symptoms related to physical fatigue and quality of life. In addition, future studies should also evaluate in this disease-specific setting some common treatment complications of RLS (i.e., tolerance, loss of efficacy, augmentation, impulse control disorders, mood disorders, and weight gain) (9) and their possible impact on clinical symptoms of PPS.

## Conflict of Interest Statement

The authors declare that the research was conducted in the absence of any commercial or financial relationships that could be construed as a potential conflict of interest.
